# Characterizing the Qatar advanced-phase SARS-CoV-2 epidemic

**DOI:** 10.1038/s41598-021-85428-7

**Published:** 2021-03-18

**Authors:** Laith J. Abu-Raddad, Hiam Chemaitelly, Houssein H. Ayoub, Zaina Al Kanaani, Abdullatif Al Khal, Einas Al Kuwari, Adeel A. Butt, Peter Coyle, Andrew Jeremijenko, Anvar Hassan Kaleeckal, Ali Nizar Latif, Robert C. Owen, Hanan F. Abdul Rahim, Samya A. Al Abdulla, Mohamed G. Al Kuwari, Mujeeb C. Kandy, Hatoun Saeb, Shazia Nadeem N. Ahmed, Hamad Eid Al Romaihi, Devendra Bansal, Louise Dalton, Mohamed H. Al-Thani, Roberto Bertollini

**Affiliations:** 1 Infectious Disease Epidemiology Group, Weill Cornell Medicine-Qatar, Cornell University, Qatar Foundation – Education City, P.O. Box 24144, Doha, Qatar; 2 World Health Organization Collaborating Centre for Disease Epidemiology Analytics On HIV/AIDS, Sexually Transmitted Infections, and Viral Hepatitis, Weill Cornell Medicine–Qatar, Cornell University, Qatar Foundation – Education City, Doha, Qatar; 3 Department of Population Health Sciences, Weill Cornell Medicine, Cornell University, New York, NY USA; 4 Department of Mathematics, Statistics, and Physics, Qatar University, Doha, Qatar; 5 Hamad Medical Corporation, Doha, Qatar; 6 College of Health Sciences, QU Health, Qatar University, Doha, Qatar; 7 Primary Health Care Corporation, Doha, Qatar; 8 Ministry of Public Health, Doha, Qatar

**Keywords:** Viral infection, Epidemiology

## Abstract

The overarching objective of this study was to provide the descriptive epidemiology of the severe acute respiratory syndrome coronavirus 2 (SARS-CoV-2) epidemic in Qatar by addressing specific research questions through a series of national epidemiologic studies. Sources of data were the centralized and standardized national databases for SARS-CoV-2 infection. By July 10, 2020, 397,577 individuals had been tested for SARS-CoV-2 using polymerase-chain-reaction (PCR), of whom 110,986 were positive, a positivity cumulative rate of 27.9% (95% CI 27.8–28.1%). As of July 5, case severity rate, based on World Health Organization (WHO) severity classification, was 3.4% and case fatality rate was 1.4 per 1,000 persons. Age was by far the strongest predictor of severe, critical, or fatal infection. PCR positivity of nasopharyngeal/oropharyngeal swabs in a national community survey (May 6–7) including 1,307 participants was 14.9% (95% CI 11.5–19.0%); 58.5% of those testing positive were asymptomatic. Across 448 ad-hoc testing campaigns in workplaces and residential areas including 26,715 individuals, pooled mean PCR positivity was 15.6% (95% CI 13.7–17.7%). SARS-CoV-2 antibody prevalence was 24.0% (95% CI 23.3–24.6%) in 32,970 residual clinical blood specimens. Antibody prevalence was only 47.3% (95% CI 46.2–48.5%) in those who had at least one PCR positive result, but 91.3% (95% CI 89.5–92.9%) among those who were PCR positive > 3 weeks before serology testing. Qatar has experienced a large SARS-CoV-2 epidemic that is rapidly declining, apparently due to growing immunity levels in the population.

## Introduction

Qatar is an Arabian Gulf country of 2.8 million people that has been affected by the severe acute respiratory syndrome coronavirus 2 (SARS-CoV-2) pandemic. The first documented case of SARS-CoV-2 community transmission was identified on March 6, 2020 and its source was linked soon after to a cluster of over 300 infections among expatriate craft and manual workers (CMW) living in high-density housing accommodations. Using mathematical modelling, the cluster size suggested the infection may have been circulating for at least 4 weeks prior to cluster identification. Restrictive social and physical distancing and other public health measures were immediately imposed in the whole country. Following the World Health Organization (WHO) guidelines, Qatar adopted a “testing, tracing, and isolation” approach, as the backbone of the national response^1^, implementing a country-wide active contact tracing and testing using real-time polymerase chain reaction (PCR). By July 11, 2020, a total of 409,199 PCR tests had been conducted for SARS-COV-2 at a rate of 145,736 per million population—one of the highest worldwide^2^. As of that date, 103,128 SARS-CoV-2 infections had been PCR-laboratory-confirmed, at a rate of 36,729 per million population—also one of the highest worldwide^2^, however only 147 COVID-19 deaths per WHO criteria^3^ had been recorded.

Qatar has a unique demographic and residential dwellings structure^4^ that proved critical in understanding SARS-CoV-2 epidemiology. Of the total population^4^, 89% are expatriates from over 150 countries^5–7^, most of whom live in the capital city, Doha^4^. About 60% of the population consists of CMW, typically working in mega-development projects^8^. This “labor” population is predominantly young (20–49 years of age), male, and single, living generally in communal shared housing accommodations^9^. The remaining 40% of the population constitutes the “urban” population, which consists of family households or discrete-unit housings that include children, adults, and elderly, with adults often working in professional or service sector jobs, either private or governmental. Overall, including the CMW, Qatar’s population is predominantly young with only 2% being > 60 years of age^5^.

Females account for only a quarter of the total population and for the vast majority are part of the urban population^5^. By nationality, Indians (28%), Bangladeshis (13%), and Nepalese (13%) are the most populous groups followed by Qataris (11%), Egyptians (9%), and Filipinos (7%)^7^. Nearly all Bangladeshis and Nepalese are in the labor population. Meanwhile, Indians, Egyptians, and Filipinos are distributed among the labor and urban populations, but with most Indians in the labor population and most Filipinos in the urban population^8^. Much of the remaining nationalities, along with Qataris, are part of the urban population^8^.

In this article, we report and synthesize the main findings of a series of national epidemiologic studies conducted with the overarching objective of providing the descriptive epidemiology of the SARS-CoV-2 epidemic in Qatar. We specifically addressed the following research questions: (1) how did SARS-CoV-2 positivity in Qatar evolve over time and what factors were associated with acquiring the infection? (2) What proportion of infections was asymptomatic and what was the extent of infection spread in the population? (3) Were there discernable patterns in PCR positivity across random testing campaigns conducted in different settings? (4) What factors were associated with having detectable antibodies against this infection? (5) What factors were associated with experiencing a severe, critical, or fatal infection? (6) How did the crude case severity rate and crude case fatality rate evolve over the course of the epidemic?

## Materials and methods

The above specific research questions were addressed through analysis of: (1) the national SARS-CoV-2 PCR testing and hospitalization database, (2) community PCR testing surveys for current infection, (3) surveillance PCR testing campaigns in workplaces and residential areas, (4) serological testing for antibody on blood specimens collected for routine clinical screening/management, (5) national Coronavirus Disease 2019 (COVID-19) death registry, and (6) SARS-CoV-2 case severity and mortality rates.

## Sources of data

### National SARS-CoV-2 PCR testing and hospitalization database

A national SARS-CoV-2 PCR testing and hospitalization database—a centralized and standardized database of all SARS-CoV-2 infections—is compiled at Hamad Medical Corporation (HMC), the main public healthcare provider and the nationally-designated provider for COVID-19 healthcare needs. The database comprises information on PCR testing conducted from February 5-July 10, 2020, including testing of suspected SARS-CoV-2 cases and traced contacts, in addition to infection surveillance testing. The database also includes data on hospital admission of COVID-19 patients and WHO severity classification for each infection^10^.

### Community surveys

A cross-sectional community survey was conducted on May 6–7, 2020 to assess SARS-CoV-2 infection levels by PCR testing in the wider population of Qatar. Recruitment sampling frame was a database of 1,461,763 registered users (mostly covering the urban population) across Qatar’s 27 Primary Health Care Corporation (PHCC) centers. A sample size of 1,118 was needed to detect a population prevalence of 3% with a margin of error of 1% using the formula ${\text{sample size}} = \frac{{z_{\alpha /2}^{2} \times prevalence \times \left( {1 - prevalence} \right)}}{{\left( {\text{margin of error}} \right)^{2} }}$^11^. To account for non-response, a phone-message invite was randomly sent to 3,120 individuals inviting them for voluntary PCR testing and collection of socio-demographic and health-related information at a drive-through set-up in any of three designated PHCC centers located close to the Central, Northern, and Western regions of Qatar. Since the start of the epidemic, PHCC centers were designated as testing facilities for suspected infection cases.

Individuals were eligible to participate if they had not been previously diagnosed with SARS-CoV-2 infection, were 10–74 years of age, and registered users of PHCC. Demographic, exposure, and symptoms data were collected on iPads using one-to-one structured interviews conducted by trained interviewers. Printed copies were available for back-up. The interview schedule was administered in English or Arabic, depending on the respondent’s preference. Nasopharyngeal and oropharyngeal swabs were collected by trained medical personnel. The survey design and the interview schedule were informed by WHO guidelines^12^ and a SARS-CoV-2 population-based survey in Iceland^13^ (Supplementary Text S1). When only 268 invitees (24.0% of required sample size) responded to the invitation, recruitment was extended to the community by open invitation in national and social media for voluntary participation in the study by visiting the designated PHCC centers. For the informed consent, a link was sent through the phone-message invite referring the participants to an electronic consent form that they can approve online. Hard copy consent forms were also made available for those who did not confirm participation ahead of time through the link. For those who were recruited through the open invitation, a verbal consent was obtained.

We further report the results of two other limited-scope community PCR testing surveys conducted among single CMW in a lockdown zone of Doha, where the first cluster of infections was identified. Residents in randomly selected blocks and housing accommodations of the industrial zone were approached for voluntary participation in PCR testing. In the first survey conducted between March 22–27, 2020, 5,120 individuals were sampled and tested using nasopharyngeal and oropharyngeal swabs collected by trained medical personnel. In a second survey in the same community on April 6–7, 2020, 886 individuals were sampled and tested following the same protocol. While these two surveys were intended to be based on probability-based sampling, logistical challenges forced a pragmatic, yet still heterogeneous by location, convenience sampling. A verbal informed consent was collected from all participants.

### Ad-hoc testing campaigns in workplaces and in residential areas

A national SARS-CoV-2 testing database was compiled including the individual-level PCR testing outcomes of 390 ad-hoc testing campaigns of 22,834 individuals invited randomly to participate in a variety of workplaces in different economic sectors, and the outcomes of 58 ad-hoc testing campaigns covering 3,881 individuals invited randomly to participate in a variety of residential areas (often where CMW live). These testing campaigns were initiated by the Ministry of Public Health (MOPH) shortly after the identification of the first cluster in March of 2020 but accelerated in subsequent weeks. The available database covers all testing conducted up to June 4, 2020.

### Seroprevalence survey

SARS-CoV-2 serological testing for antibody was performed on a convenience sample of residual blood specimens collected for routine clinical screening or clinical management from 32,970 outpatient and inpatient departments at HMC for a variety of health conditions, between May 12-July 12, 2020. Specimens were unlinked of identifying information about prior PCR testing before blood collection. The sample under-represented the CMW population as they receive outpatient healthcare primarily at customized healthcare centers operated by the Qatar Red Crescent Society. The database was subsequently linked to the national SARS-CoV-2 PCR testing and hospitalization database to conduct additional analyses linking PCR and antibody test results. A waiver of informed consent was approved by the Institutional Review Boards for this study since analysis was conducted retrospectively on existing residual blood specimens.

### National COVID-19 mortality database

COVID-19 deaths were extracted from a national COVID-19 mortality validation database, a centralized registry of COVID-19-related deaths per WHO classification^3^, compiled at HMC. The database comprises information on COVID-19 deaths from February 26-July 10, 2020.

## Laboratory methods

All PCR testing was conducted at HMC Central Laboratory, the national reference laboratory, or at Sidra Medicine Laboratory, following standardized protocols. Nasopharyngeal and/or oropharyngeal swabs (Huachenyang Technology, China) were collected and placed in Universal Transport Medium (UTM). Aliquots of UTM were: extracted on the QIAsymphony platform (QIAGEN, USA) and tested with real-time reverse-transcription PCR (RT-qPCR) using the TaqPath COVID-19 Combo Kit (Thermo Fisher Scientific, USA) on a ABI 7500 FAST (ThermoFisher, USA); extracted using a custom protocol^14^ on a Hamilton Microlab STAR (Hamilton, USA) and tested using the AccuPower SARS-CoV-2 Real-Time RT-PCR Kit (Bioneer, Korea) on a ABI 7500 FAST; or loaded directly to a Roche cobas 6800 system and assayed with the cobas SARS-CoV-2 Test (Roche, Switzerland). The first assay targets the S, N, and ORF1ab regions of the virus; the second targets the virus’ RdRp and E-gene regions; and the third targets the ORF1ab and E-gene regions.

Serological testing was performed using the Roche Elecsys Anti-SARS-CoV-2 (Roche, Switzerland), an electrochemiluminescence immunoassay that uses a recombinant protein representing the nucleocapsid (N) antigen for the determination of antibodies against SARS-CoV-2. Qualitative anti-SARS-CoV-2 results were generated following the manufacturer’s instructions^15^ (reactive: cutoff index ≥ 1.0 vs. non-reactive: cutoff index < 1.0).

### Statistical analysis

Frequency distributions were generated to describe the demographic/clinical profile of tested individuals. Where applicable, probability weights were applied to adjust for unequal sample selection using the Qatar population distribution by sex, age group, and nationality^5,7,16^. Chi-square test and univariable logistic regressions were implemented to explore associations. Odds ratios (ORs), 95% confidence intervals (CIs), and *p* values were reported. Covariates with *p* value ≤ 0.1 in univariable regression analysis were considered possibly associated with the outcome variables, and were thus included in the multivariable analysis for estimation of adjusted odds ratios (AORs) and associated 95% CIs and *p* values. Covariates with *p* value ≤ 0.05 in the multivariable model were considered as predictors of the outcome.

Where relevant, the pooled mean for SARS-CoV-2 PCR positivity was estimated using random-effects meta-analysis. To this end, variances of measures were first stabilized using a Freeman-Tukey type arcsine square-root transformation^17,18^. Measures were then weighted using the inverse-variance method^18,19^, prior to being pooled using a DerSimonian-Laird random-effects model^20^. Factors associated with higher PCR positivity and sources of between-study heterogeneity were then identified using random-effects meta-regression, applying the same criteria used for conventional regression analysis (described above).

Time was factored in different analyses given interest in assessing the temporal trend in different outcomes, as well as to control for time as an important confounder in both testing PCR positive and experiencing infection severity throughout the epidemic’s evolution.

### Ethics declarations

All methods were carried out in accordance with relevant guidelines and regulations. Studies were approved by Hamad Medical Corporation and Weill Cornell Medicine-Qatar Institutional Review Boards.

## Results

### Analysis of the national SARS-CoV-2 PCR testing and hospitalization database

By July 10, a total of 397,577 individuals had been tested for current infection (14.2% of the population of Qatar), of whom 110,986 were PCR positive for SARS-CoV-2 (4.0% of the population), for an overall cumulative positivity rate of 27.9% (95% CI 27.8–28.1%). Positivity rate increased rapidly starting from March and peaked at 45.1% on May 22, after which it has been declining and was 17.8% on July 9.

Adjusted odds of PCR positivity were 1.6-fold (95% CI 1.5–1.6) higher in males compared to females (Table 1). Odds of PCR positivity varied by nationality and were highest among Nepalese (AOR: 4.5; 95% CI 4.3–4.6) and Bangladeshis (AOR: 3.9; 95% CI 3.8–4.0) and lowest among Qataris (AOR: 0.57; 95% CI 0.55–0.59), compared to other nationalities.

**Table 1:** Associations with current infection in Qatar based on analysis of the national SARS-CoV-2 PCR testing and hospitalization database.

Characteristics	Sample size	SARS-CoV-2 positive		Univariable analysis		Multivariable analysis	
	N (%)	N (%)	*p* value	OR (95% CI)	*p* value *	AOR (95% CI)	*p* value^†^
**Sex**							
Females	102,260 (25.7)	16,813 (16.4)	< 0.001	1.00		1.00	
Males	295,317 (74.3)	94,173 (31.9)		2.38 (2.34–2.42)	< 0.001	1.57 (1.54–1.60)	< 0.001
**Age (years)**							
< 10	22,161 (5.6)	4,851 (21.9)	< 0.001	1.00		1.00	
10–19	16,158 (4.1)	3,819 (23.6)		1.10 (1.05–1.16)	< 0.001	1.34 (1.27–1.41)	< 0.001
20–29	95,973 (24.1)	27,038 (28.2)		1.40 (1.35–1.45)	< 0.001	0.77 (0.74–0.80)	< 0.001
30–39	137,367 (34.6)	39,660 (28.9)		1.45 (1.40–1.50)	< 0.001	0.74 (0.71–0.77)	< 0.001
40–49	73,616 (18.5)	22,698 (30.8)		1.59 (1.54–1.65)	< 0.001	0.83 (0.80–0.86)	< 0.001
50–59	35,256 (8.9)	9,541 (27.1)		1.32 (1.27–1.38)	< 0.001	0.84 (0.81–0.88)	< 0.001
60–69	12,812 (3.2)	2,724 (21.3)		0.96 (0.91–1.02)	0.169	0.87 (0.82–0.92)	< 0.001
70–79	3,126 (0.8)	520 (16.6)		0.71 (0.64–0.79)	< 0.001	0.91 (0.82–1.01)	0.092
80 +	1,108 (0.3)	135 (12.2)		0.50 (0.41–0.59)	< 0.001	0.74 (0.62–0.90)	0.002
**Nationality**							
All other nationalities^‡^	66,472 (16.7)	11,218 (16.9)	< 0.001	1.00		1.00	
Indian	89,216 (22.4)	31,931 (35.8)		2.75 (2.68–2.81)	< 0.001	2.55 (2.49–2.62)	< 0.001
Bangladeshi	34,201 (8.6)	15,312 (44.8)		3.99 (3.88–4.11)	< 0.001	3.91 (3.79–4.04)	< 0.001
Nepalese	41,676 (10.5)	20,162 (48.4)		4.62 (4.49–4.75)	< 0.001	4.46 (4.33–4.60)	< 0.001
Pakistani	18,982 (4.8)	6,758 (35.6)		2.72 (2.63–2.82)	< 0.001	2.46 (2.38–2.56)	< 0.001
Sudanese	11,559 (2.9)	2,305 (19.9)		1.23 (1.17–1.29)	< 0.001	1.15 (1.09–1.21)	< 0.001
Sri Lankan	11,583 (2.9)	4,191 (36.2)		2.79 (2.68–2.92)	< 0.001	2.73 (2.61–2.86)	< 0.001
Egyptian	18,312 (4.6)	5,057 (27.6)		1.88 (1.81–1.95)	< 0.001	1.72 (1.65–1.79)	< 0.001
Filipino	35,094 (8.8)	6,505 (18.5)		1.12 (1.08–1.16)	< 0.001	1.24 (1.19–1.28)	< 0.001
Qatari	70,482 (17.7)	7,547 (10.7)		0.59 (0.57–0.61)	< 0.001	0.57 (0.55–0.59)	< 0.001
**Time**							
05 Feb–21 Mar	9,061 (2.3)	658 (7.3)	< 0.001	1.00		1.00	
22 Mar–28 Mar	7,090 (1.8)	450 (6.3)		0.87 (0.76–0.98)	0.023	0.72 (0.63–0.81)	< 0.001
29 Mar–04 Apr	7,622 (1.9)	860 (11.3)		1.62 (1.46–1.81)	< 0.001	2.53 (2.27–2.82)	< 0.001
05 Apr–11 Apr	8,030 (2.0)	1,158 (14.4)		2.15 (1.95–2.38)	< 0.001	2.45 (2.21–2.72)	< 0.001
12 Apr–18 Apr	11,013 (2.8)	2,796 (25.4)		4.35 (3.97–4.76)	< 0.001	4.53 (4.13–4.96)	< 0.001
19 Apr–25 Apr	16,521 (4.2)	4,961 (30.0)		5.48 (5.03–5.97)	< 0.001	5.23 (4.79–5.71)	< 0.001
26 Apr–02 May	16,600 (4.2)	4,982 (30.0)		5.48 (5.02–5.97)	< 0.001	5.86 (5.37–6.40)	< 0.001
03 May–09 May	21,962 (5.5)	7,113 (32.4)		6.12 (5.62–6.65)	< 0.001	6.36 (5.84–6.93)	< 0.001
10 May–16 May	28,526 (7.2)	10,265 (36.0)		7.18 (6.61–7.80)	< 0.001	8.08 (7.43–8.80)	< 0.001
17 May–23 May	31,428 (7.9)	11,396 (36.3)		7.27 (6.69–7.89)	< 0.001	7.94 (7.30–8.64)	< 0.001
24 May–30 May	34,280 (8.6)	12,883 (37.6)		7.69 (7.08–8.35)	< 0.001	8.60 (7.91–9.35)	< 0.001
31 May–6 Jun	35,215 (8.9)	11,634 (33.0)		6.30 (5.80–6.84)	< 0.001	7.63 (7.02–8.30)	< 0.001
07 Jun–13 Jun	36,636 (9.2)	10,968 (29.9)		5.46 (5.03–5.93)	< 0.001	6.43 (5.91–7.00)	< 0.001
14 Jun–20 Jun	36,063 (9.1)	9,936 (27.6)		4.86 (4.47–5.27)	< 0.001	5.93 (5.45–6.46)	< 0.001
21 Jun–27 Jun	38,015 (9.6)	9,726 (25.6)		4.39 (4.04–4.77)	< 0.001	5.49 (5.05–5.98)	< 0.001
28 Jun–04 Jul	36,991 (9.3)	7,831 (21.2)		3.43 (3.16–3.73)	< 0.001	4.07 (3.74–4.43)	< 0.001
05 Jul–10 Jul	22,524 (5.7)	3,369 (15.0)		2.25 (2.06–2.45)	< 0.001	2.76 (2.53–3.02)	< 0.001

*AOR* adjusted odds ratio, *CI* confidence interval, *OR* odds ratio, *PCR* polymerase chain reaction.

*Covariates with *p* value ≤ 0.1 in the univariable analysis were included in the multivariable analysis.

^†^Covariates with *p* value ≤ 0.05 in the multivariable analysis were considered predictors of SARS-CoV-2 current infection.

^‡^These include all other nationalities residing in Qatar.

A time trend was observed with odds of PCR positivity gradually increasing (Table 1), consistent with an exponentially growing epidemic, to reach 8.6-fold (95% CI 7.9–9.4) higher in May 24–30 compared to the beginning of the epidemic, but rapidly declining thereafter to be only 2.8-fold (95% CI 2.5–3.0) higher in July 5–10.

### PCR positivity in community surveys

A total of 1,307 individuals participated in the PCR community survey conducted on May 6–7 (Table 2). There were differences in age, nationality, and educational attainment between the participants who were randomly invited and those who participated through the open announcement, but the differences were not major (Supplementary Table S1). No differences were observed by sex or occupation.

**Table 2:** Results of the community survey conducted on May 6–7, 2020 and associations with PCR positivity in Qatar.

Characteristics	Original sample size	SARS-CoV-2 positive*		Univariable regression analysis		Multivariable regression analysis	
	N (%)	N (%^†^)	*p* value	OR (95% CI)^‡^	*p* value^§^	AOR (95% CI)^‡^	*p* value^¶^
**Socio-demographic characteristics**							
*Sex*							
Females	272 (20.8)	21 (10.6)	0.318	1.00		–	–
Males	1,035 (79.2)	135 (15.9)		1.59 (0.64–3.97)	0.322	–	–
*Age (years)*							
< 30	320 (24.5)	41 (13.7)	0.811	1.00		–	–
30–39	534 (40.9)	76 (15.9)		1.19 (0.64–2.24)	0.582	–	–
40–49	264 (20.2)	28 (17.3)		1.32 (0.53–3.30)	0.549	–	–
50 +	189 (14.5)	11 (10.5)		0.74 (0.18–3.07)	0.679	–	–
*Nationality*							
All other nationalities^#^	255 (19.5)	16 (6.2)	0.019	1.00		1.00	
Indian	531 (40.6)	86 (17.4)		3.18 (1.80–5.64)	< 0.001	2.61 (1.20–5.68)	0.016
Filipino	116 (8.9)	19 (17.4)		3.18 (1.53–6.60)	0.002	3.23 (1.28–8.14)	0.013
Sri Lankan	32 (2.5)	10 (33.4)		7.56 (2.96–19.32)	< 0.001	5.76 (1.88–17.63)	0.002
Egyptian	90 (6.9)	7 (8.5)		1.40 (0.55–3.60)	0.481	1.80 (0.64–5.04)	0.265
Nepalese	27 (2.1)	6 (24.6)		4.92 (1.67–14.53)	0.004	5.21 (1.54–17.55)	0.008
Bangladeshi	14 (1.1)	3 (21.2)		4.06 (0.99–16.57)	0.051	1.66 (0.46–6.00)	0.441
Pakistani	23 (1.8)	4 (17.3)		3.16 (0.93–10.73)	0.066	2.37 (0.53–10.62)	0.259
Sudanese	20 (1.5)	2 (12.4)		2.14 (0.43–10.55)	0.351	2.10 (0.51–8.72)	0.306
Qatari	199 (15.2)	3 (1.9)		0.29 (0.08–1.09)	0.068	0.37 (0.10–1.40)	0.141
*Educational attainment*							
Bachelor’s degree	511 (39.1)	52 (10.7)	0.038	1.00		1.00	
High school	440 (33.7)	59 (13.7)		1.34 (0.73–2.44)	0.346	1.17 (0.60–2.28)	0.645
Post-graduate studies	118 (9.0)	16 (30.5)		3.68 (1.41–9.57)	0.008	5.36 (2.29–12.56)	< 0.001
Unknown	238 (18.2)	29 (15.1)		1.50 (0.70–3.21)	0.299	0.70 (0.21–2.38)	0.571
Occupation**							
Healthcare	48 (3.7)	2 (1.8)	0.011	1.00		1.00	
Aviation/Police/Government	57 (4.4)	8 (22.4)		15.67 (2.51–97.89)	0.003	18.82 (2.46–144.22)	0.005
Hospitality/Retail/Education	118 (9.0)	6 (3.8)		2.16 (0.38–12.14)	0.381	1.51 (0.18–12.39)	0.703
Professional/Office	292 (22.3)	26 (10.2)		6.17 (1.28–29.78)	0.024	6.82 (1.10–42.18)	0.039
Transportation/driver	98 (7.5)	17 (28.7)		21.82 (3.98–119.60)	< 0.001	15.04 (2.22–101.73)	0.005
Construction/Blue collar jobs	213 (16.3)	37 (18.4)		12.18 (2.41–61.52)	0.003	11.95 (1.98–72.03)	0.007
Not employed	141 (10.8)	16 (9.2)		5.46 (1.10–27.05)	0.038	7.06 (0.98–50.66)	0.052
Unknown/Other	340 (26.0)	44 (17.1)		11.14 (3.01–93.46)	0.003	16.29 (2.50–106.31)	0.004
**Study-related characteristics**							
*Study recruitment/invitation*							
Invited/sampled	268 (20.5)	17 (10.9)	0.441	1.00		–	–
In same car/invited	145 (11.1)	20 (11.6)		1.08 (0.37–3.15)	0.894	–	–
Not invited	687 (52.6)	92 (17.3)		1.71 (0.63–4.63)	0.292	–	–
Unknown	207 (15.8)	27 (13.7)		1.30 (0.44–3.80)	0.636	–	–
*Primary Health Care Corporation center*							
Leabib/Unknown	304 (23.3)	13 (6.5)	0.075	1.00		1.00	
Al-Thumama	567 (43.4)	86 (17.1)		2.96 (1.00 -8.80)	0.050	1.38 (0.41–4.65)	0.601
Al-Waab	436 (33.4)	57 (17.5)		3.05 (1.05–8.89)	0.041	2.10 (0.64–6.91)	0.222
**Clinical characteristics**							
*Presence of symptoms*							
No symptoms	849 (65.0)	72 (11.6)	< 0.001	1.00		1.00	
One symptom	139 (10.6)	18 (19.4)		1.84 (0.64–5.30)	0.258	1.49 (0.61–3.68)	0.382
Two symptoms	80 (6.1)	19 (19.4)		1.84 (0.75–4.47)	0.180	2.28 (0.90–5.79)	0.081
Three or more symptoms	68 (5.2)	24 (50.3)		7.73 (2.97–20.08)	< 0.001	6.04 (2.56–14.24)	< 0.001
Unknown	171 (13.1)	23 (13.6)		1.21 (0.58–2.53)	0.620	1.04 (0.28–3.83)	0.950
*Sought medical attention in the past 2 weeks*							
No	1,125 (86.1)	130 (14.2)	< 0.001	1.00		1.00	
Yes	11 (0.8)	3 (69.8)		14.03 (2.15–91.51)	0.006	9.84 (1.55–62.64)	0.016
Unknown	171 (13.1)	23 (13.6)		0.96 (0.48–1.91)	0.900	Omitted	–
*Hospitalized in the past 2 weeks*							
No	1,133 (99.7)	132 (15.1)	0.784	1.00		–	–
Yes	3 (0.3)	1 (25.7)		1.95 (0.17–22.02)	0.590	–	–
Unknown	171 (13.1)	23 (13.6)		0.89 (0.44–1.78)	0.738	–	–
*Contact with index case*							
No	750 (57.4)	82 (15.2)	0.961	1.00		–	–
Yes	281 (21.5)	42 (14.9)		0.98 (0.54–1.76)	0.943	–	–
Unknown (do not know)	276 (21.1)	32 (14.2)		0.93 (0.44–1.94)	0.844	–	–
**SARS-CoV-2 PCR positivity**							
*Test result*							
Negative	1,125 (86.1)	–	–	–	–	–	–
Positive	156 (11.9)	156 (14.9)	–	–	–	–	–
Inconclusive	26 (2.0)	–	–	–	–	–	–

*AOR* adjusted odds ratio, *CI* confidence interval, *OR* odds ratio, *PCR* polymerase chain reaction.

*Only 1,281 samples with confirmed results were analyzed.

^†^Row percentages weighted by age and nationality.

^‡^Estimates are weighted by age and nationality.

^§^Covariates with *p* value ≤ 0.1 in the univariable analysis were included in the multivariable analysis.

^¶^Covariates with *p* value ≤ 0.05 in the multivariable analysis were considered predictors of SARS-CoV-2 infection.

^#^These include all other nationalities residing in Qatar.

**Occupation categories were grouped together based on epidemiological relevance, that is to factor the frequency of social contacts (such as for the aviation sector and the police) or the effect of social and physical distancing restrictions (such as for hospitality, retail, and education sectors).

A total of 156 persons tested PCR positive. The weighted (for total population of Qatar) SARS-CoV-2 PCR prevalence was 14.9% (95% CI 11.5–19.0%). After controlling for confounders, strong evidence for an association with positivity (*p* value ≤ 0.05) was found for nationality, educational attainment, occupation, presence of symptoms, and seeking medical attention, but not for sex, age, study recruitment/invitation, PHCC center location, seeking hospitalization, or history of contact with an index case (Table 2). The differences were pronounced for nationality, occupation, presence of symptoms, and seeking medical attention.

The proportion of asymptomatic individuals was 58.5% in those testing positive and 79.5% in those testing negative (*p* < 0.001; Table 3). While there was no evidence for an association with reporting one or two symptoms, reporting three or more symptoms was significantly associated with SARS-CoV-2 infection (Table 3). Associations with specific symptoms are summarized in Supplementary Table S2—infection was significantly associated with fever, fatigue, muscle ache, other respiratory symptoms, nausea/vomiting, loss of sense of smell, and loss of sense of taste. However, < 9.5% of those positive reported each of these individual symptoms apart from fever which was reported by 28.6% of those positive.

**Table 3:** Comparison of individuals who tested PCR negative versus individuals who tested PCR positive for SARS-CoV-2 infection* in the community survey conducted on May 6–7, 2020 with respect to presence of symptoms.

Reported symptoms	SARS-CoV-2 negative	SARS-CoV-2 positive		Univariable regression analysis	
	N (%^†^)	N (%^†^)	*p* value	OR (95% CI)^‡^	*p* value
**Presence of symptoms**					
Asymptomatic	764 (79.5)	72 (58.5)	0.003	1.00	
Symptomatic	217 (20.5)	61 (41.6)		2.75 (1.39–5.45)	0.004
**Overall**					
No symptoms	764 (79.5)	72 (58.5)	< 0.001	1.00	
One symptom	119 (11.0)	18 (14.9)		1.84 (0.64–5.30)	0.258
Two symptoms	59 (6.3)	19 (8.5)		1.84 (0.75–4.47)	0.180
Three or more symptoms	39 (3.2)	24 (18.1)		7.73 (2.97–20.08)	< 0.001

*CI* confidence interval, *OR* odds ratio, *PCR* polymerase chain reaction.

*Only 1,114 samples with information on symptoms and confirmed results were analyzed.

^†^Column percentages weighted by age and nationality.

^‡^Estimates are weighted by age and nationality.

An analysis of PCR cycle threshold (Ct) values’ association with presence of symptoms was performed with the understanding that PCR positivity does not only reflect recent active infection, but also time after recovery, as PCR tests are sensitive enough to pick even non-viable viral fragments even for weeks following recovery from active infection^21,22^. Supplementary Table S3 shows the association between PCR Ct value and presence of symptoms. Odds of having a Ct value < 25 (proxy for recent infection^23^) were five-fold higher among symptomatic compared to asymptomatic individuals.

In the two other (limited-scope) community surveys conducted among single CMW in a lockdown zone of Doha, where the first cluster of infections was identified, PCR positivity was first assessed at 1.4% (95% CI 1.1–1.8%) on March 22–27 in a sample comprising 5,120 individuals, and at 4.4% (95% CI 3.2–6.0%) on April 6–7 in a sample comprising 886 individuals.

### PCR positivity in the ad-hoc testing campaigns in workplaces and in residential areas

By June 4, 390 random PCR testing campaigns in workplaces and 58 in residential areas had been conducted testing 22,834 and 3,881 individuals, respectively. At least one infected individual was identified in 72.3% of workplaces and 72.4% of residential areas. The median PCR positivity in workplaces was 7.8%, while the pooled mean was 15.1% (95% CI 13.0–17.3%). Meanwhile, the median PCR positivity in residential areas was 15.3%, and the pooled mean was 20.3% (95% CI 15.4–25.6%).

Supplementary Table S4 shows the pooled mean PCR positivity across these testing campaigns and over time. The pooled mean was 17.6% (95% CI 9.3–27.7%) in March, but testing campaigns then were focused in the specific neighborhood where the first cluster was identified. Starting from April, testing was expanded throughout Qatar. PCR positivity increased steadily and rapidly consistent with exponential growth from about 5% in early April to a peak of 23% by mid to late May, after which the prevalence started declining.

Table 4 shows the results of the multivariable meta-regression analysis of PCR positivity across these testing campaigns. There was no evidence for differences in PCR positivity between the workplaces and residential areas, consistent with a widely disseminated epidemic. However, there was strong evidence for a rapidly growing epidemic from March up to the third week of May, after which the epidemic started declining.

**Table 4:** Meta-regression results to identify associations with SARS-CoV-2 PCR positivity in the random testing campaigns conducted in workplaces and in residential areas, up to June 4, 2020.

	Population groups	Tested	Univariable analysis		Multivariable analysis	
	Total N	Total n	OR (95% CI)	*p* value *	AOR (95% CI)	*p* value^†^
**Swab type**						
Workplace	347	22,834	1.00		1.00	
Residential	45	3,881	1.54 (0.94–2.53)	0.086	1.44 (0.89–2.31)	0.134
**Time**						
March–April 04–10	36^‡^	2,408	1.00		1.00	
April 11–17	28	2,476	1.38 (0.65–2.91)	0.403	1.35 (0.64–2.85)	0.435
April 18–24	56	5,422	3.25 (1.72–6.13)	< 0.001	3.16 (1.67–5.96)	< 0.001
April 25–May 01	39	2,548	3.72 (1.87–7.40)	< 0.001	3.56 (1.79–7.09)	< 0.001
May 02–08	65	4,254	2.52 (1.36–4.67)	0.003	2.51 (1.36–4.65)	0.004
May 09–15	36	3,189	4.17 (2.07–8.39)	< 0.001	4.21 (2.09–8.47)	< 0.001
May 16–22	56	3,103	4.30 (2.28–8.11)	< 0.001	4.23 (2.24–7.98)	< 0.001
May 23–29	48	2,175	2.89 (1.50–5.56)	0.002	2.89 (1.50–5.56)	0.002
May 30–June 04	28	1,140	0.74 (0.35–1.57)	0.434	0.75 (0.35–1.57)	0.440

*AOR* adjusted odds ratio, *CI* confidence interval, *OR* odds ratio, *PCR* polymerase chain reaction.

*Predictors with *p* value ≤ 0.1 were eligible for inclusion in the multivariable analysis.

^†^Predictors with *p* value < 0.05 in the multivariable model were considered statistically significant.

^‡^Includes 4 populations tested in March.

### Anti-SARS-COV-2 seropositivity

A total of 32,970 individuals were tested for SARS-CoV-2 antibodies with the blood specimens drawn between May 12-July 12, with a median day of June 28. A total of 5,448 individuals had detectable antibodies. The weighted (for total population of Qatar) antibody prevalence was 24.0% (95% CI 23.3–24.6%).

There were large differences in antibody prevalence by sex and nationality. After controlling for confounders (Table 5), adjusted odds of antibody positivity were 2.9-fold (95% CI 2.6–3.2) higher in males compared to females, and were highest among Bangladeshis (AOR: 6.8; 95% CI 5.9–8.0) and Nepalese (AOR: 6.6; 95% CI 5.7–7.8), but lowest among Qataris (AOR: 0.7; 95% CI 0.6–0.8), compared to other nationalities. Adjusted odds of antibody positivity increased incrementally with age.

**Table 5:** Results of the seroprevalence survey and associations with antibody positivity.

Characteristics	Tested	Seropositive		Univariable regression analysis		Multivariable regression analysis	
	N (%)	N (%*)	*p* value	OR* (95% CI)	*p* value^†^	AOR* (95% CI)	*p* value^‡^
**Sex**							
Females	14,366 (43.6)	958 (7.8)	< 0.001	1.00		1.00	
Males	18,604 (56.4)	4,490 (29.2)		4.89 (4.46–5.37)	< 0.001	2.92 (2.64–3.24)	< 0.001
**Age (years)**							
< 10	814 (2.5)	40 (5.5)	< 0.001	1.00		1.00	
10–19	1,222 (3.7)	70 (6.7)		1.25 (0.78–1.99)	0.356	1.80 (1.11–2.93)	0.018
20–29	4,738 (14.4)	613 (23.1)		5.22 (3.59–7.59)	< 0.001	3.35 (2.26–4.96)	< 0.001
30–39	8,437 (25.6)	1,379 (25.3)		5.87 (4.06–8.47)	< 0.001	3.31 (2.25–4.87)	< 0.001
40–49	7,513 (22.8)	1,582 (30.1)		7.47 (5.18–10.79)	< 0.001	3.82 (2.60–5.61)	< 0.001
50–59	5,455 (16.5)	1,150 (30.4)		7.57 (5.24–10.95)	< 0.001	4.27 (2.91–6.28)	< 0.001
60–69	2,982 (9.0)	452 (23.9)		5.46 (3.74–7.97)	< 0.001	3.86 (2.60–5.74)	< 0.001
70–79	1,254 (3.8)	122 (13.9)		2.79 (1.83–4.25)	< 0.001	3.60 (2.33–5.56)	< 0.001
80 +	555 (1.7)	40 (9.8)		1.88 (1.13–3.12)	0.015	3.36 (1.97–5.72)	< 0.001
**Nationality**							
All other nationalities^§^	6,776 (20.6)	479 (7.2)	< 0.001	1.00		1.00	
Indian	5,553 (16.8)	1,330 (24.3)		4.16 (3.64–4.75)	< 0.001	3.17 (2.76–3.63)	< 0.001
Bangladeshi	2,284 (6.9)	996 (44.6)		10.44 (9.03–12.09)	< 0.001	6.83 (5.86–7.96)	< 0.001
Nepalese	1,622 (4.9)	732 (44.1)		10.22 (8.76–11.92)	< 0.001	6.63 (5.65–7.77)	< 0.001
Pakistani	1,524 (4.6)	412 (23.8)		4.04 (3.36–4.85)	< 0.001	3.91 (3.24–4.73)	< 0.001
Sudanese	1,123 (3.4)	131 (11.0)		1.60 (1.23–2.08)	0.001	1.54 (1.18–2.02)	0.002
Sri Lankan	696 (2.1)	185 (26.5)		4.68 (3.74–5.87)	< 0.001	3.38 (2.70–4.23)	< 0.001
Egyptian	2,612 (7.9)	306 (11.7)		1.72 (1.44–2.07)	< 0.001	1.54 (1.28–1.85)	< 0.001
Filipino	2,224 (6.7)	465 (20.3)		3.31 (2.81–3.89)	< 0.001	3.31 (2.80–3.91)	< 0.001
Qatari	8,556 (26.0)	412 (4.7)		0.63 (0.53–0.75)	< 0.001	0.66 (0.55–0.79)	< 0.001
**Time (antibody testing)**							
12 May–31 May	937 (2.8)	264 (30.4)	< 0.001	1.00		1.00	
01 Jun–15 Jun	3,485 (10.6)	793 (30.2)		0.99 (0.81–1.21)	0.931	1.34 (1.08–1.67)	0.009
16 Jun–30 Jun	14,954 (45.4)	2,261 (22.6)		0.67 (0.56–0.80)	< 0.001	1.05 (0.86–1.28)	0.651
01 Jul–12 Jul	13,594 (41.2)	2,130 (23.1)		0.69 (0.57–0.83)	< 0.001	1.11 (0.91–1.35)	0.316

*AOR* adjusted odds ratio, *CI* confidence interval, *OR* odds ratio.

*Estimates are weighted by sex, age, and nationality.

^†^Covariates with *p* value ≤ 0.1 in the univariable analysis were included in the multivariable analysis.

^‡^Covariates with *p* value ≤ 0.05 in the multivariable analysis were considered predictors of anti-SARS-CoV-2 positivity.

^§^These include all other nationalities residing in Qatar.

Linking the antibody testing database to the national SARS-CoV-2 PCR testing and hospitalization database identified 17,062 individuals with available PCR and antibody testing results. Antibody prevalence was 6.2% (95% CI 5.7–6.7%) in those who had all PCR negative results. Antibody prevalence was 47.3% (95% CI 46.2–48.5%) in those who had at least one PCR positive result. In the remaining 15,908 individuals who were tested for detectable antibodies but had no previous PCR testing results, 1,372 were seropositive, that is an antibody prevalence of 8.6% (95% CI 8.2–9.1%).

**Figure 1:**
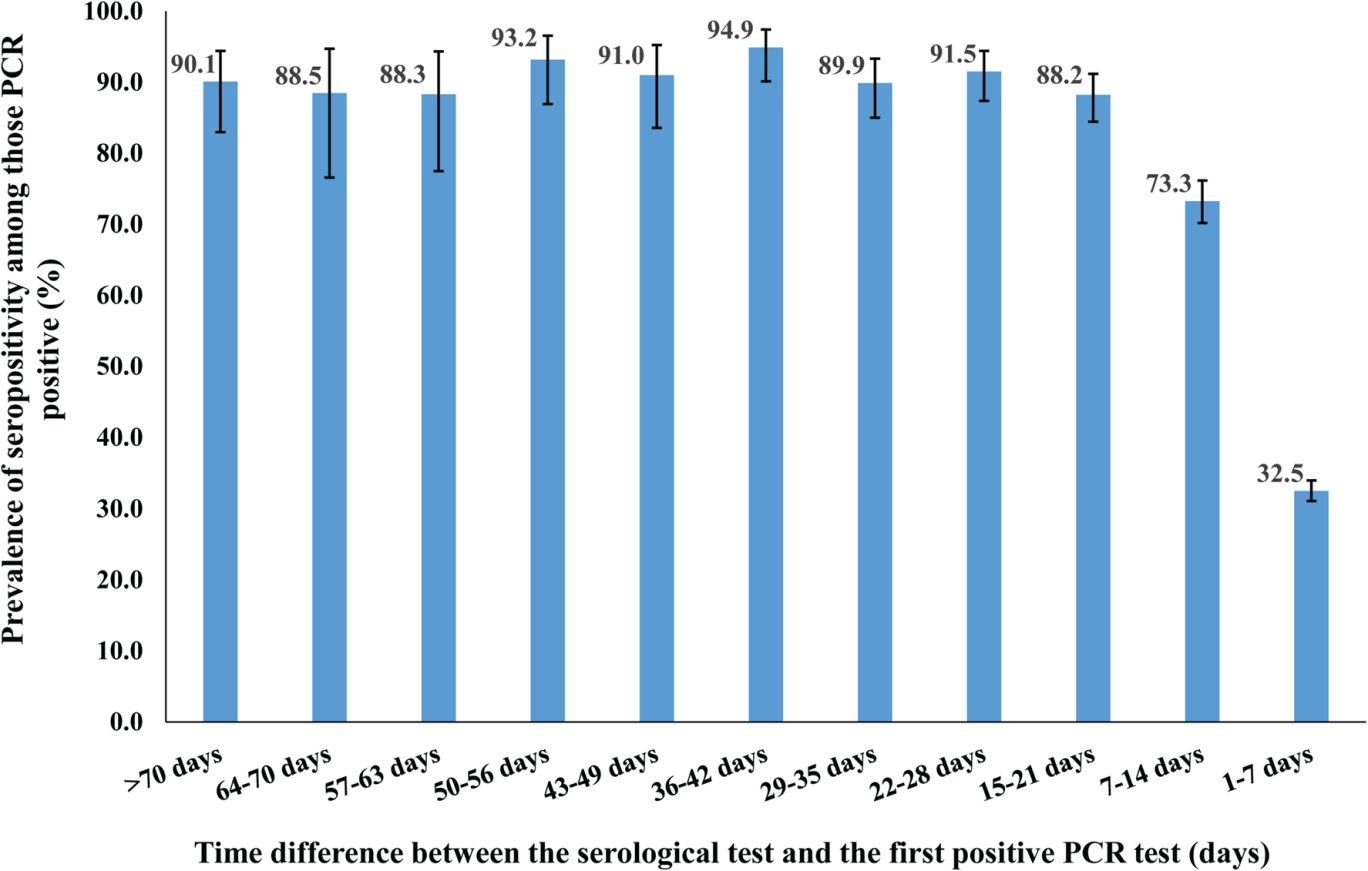
Anti-SARS-CoV-2 prevalence assessed at different time intervals for the duration between the first PCR positive test and the antibody test. *PCR* polymerase chain reaction.

Figure 1 shows antibody prevalence versus the time difference between the first positive PCR test and the antibody test. Overall, antibody positivity was > 88% among those who had their first positive PCR test > 15 days before the antibody test. Antibody positivity declined steadily the closer is the PCR test date to the antibody test date, consistent with a weeks-long delay between onset of infection and development of detectable antibodies.

### Basic socio-demographic associations with severe, critical, or fatal infection

**Figure 2:**
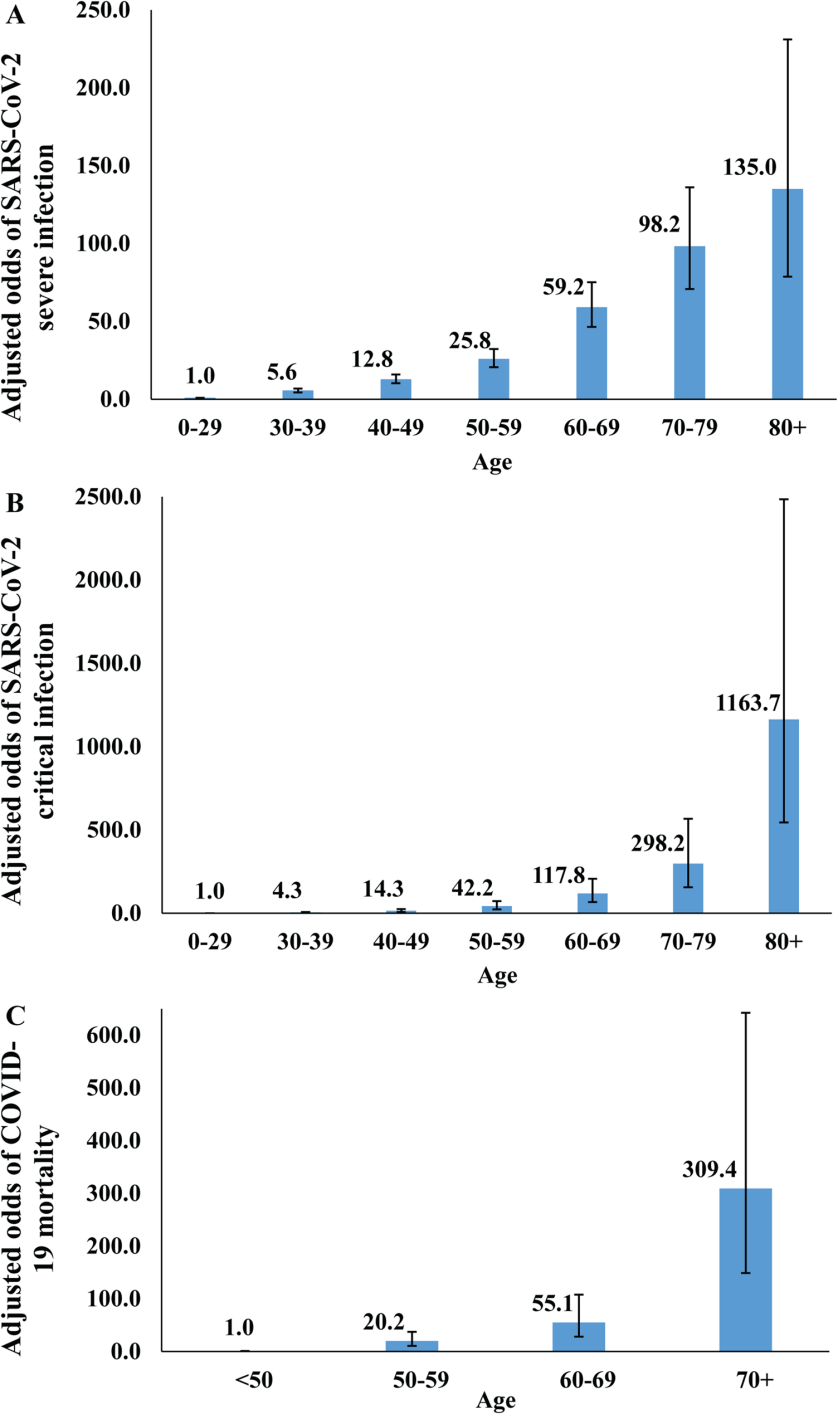
Association of age with (**A**) SARS-CoV-2 severe infection, (**B**) SARS-CoV-2 critical infection, and (**C**) COVID-19 death, adjusted for sex, nationality, and time of PCR testing. Severe infections, critical infections, and COVID-19 deaths were defined based on the World Health Organization criteria for classifying SARS-CoV-2 infection severity^10^ and COVID-19-related death^3^.

Supplementary Tables S5–S7 show basic socio-demographic associations with each of severe and critical infection and COVID-19 death (all per WHO classification)^3,10^, respectively, as of June 25. After controlling for confounders, age was, by far, the strongest predictor of each of these outcomes, and more so for critical infection and death (Fig. 2). Risk of serious disease and death increased immensely for those > 50 years of age, who are a small minority in the population of Qatar (8.8%).

Males had 1.6-fold higher odds for developing severe and critical infection compared to females (Supplementary Tables S5–S6), but no difference was observed for death, possibly because of the small number of deaths (Supplementary Table S7). Overall, there were no major differences in these outcomes by nationality, but there was evidence for higher risk of serious disease for Bangladeshis and Filipinos, and lower risk for Indians. The odds of disease and mortality declined with time, but this may just reflect the lagging time between onset of infection and disease/mortality.

### Crude case severity and fatality rates

**Figure 3:**
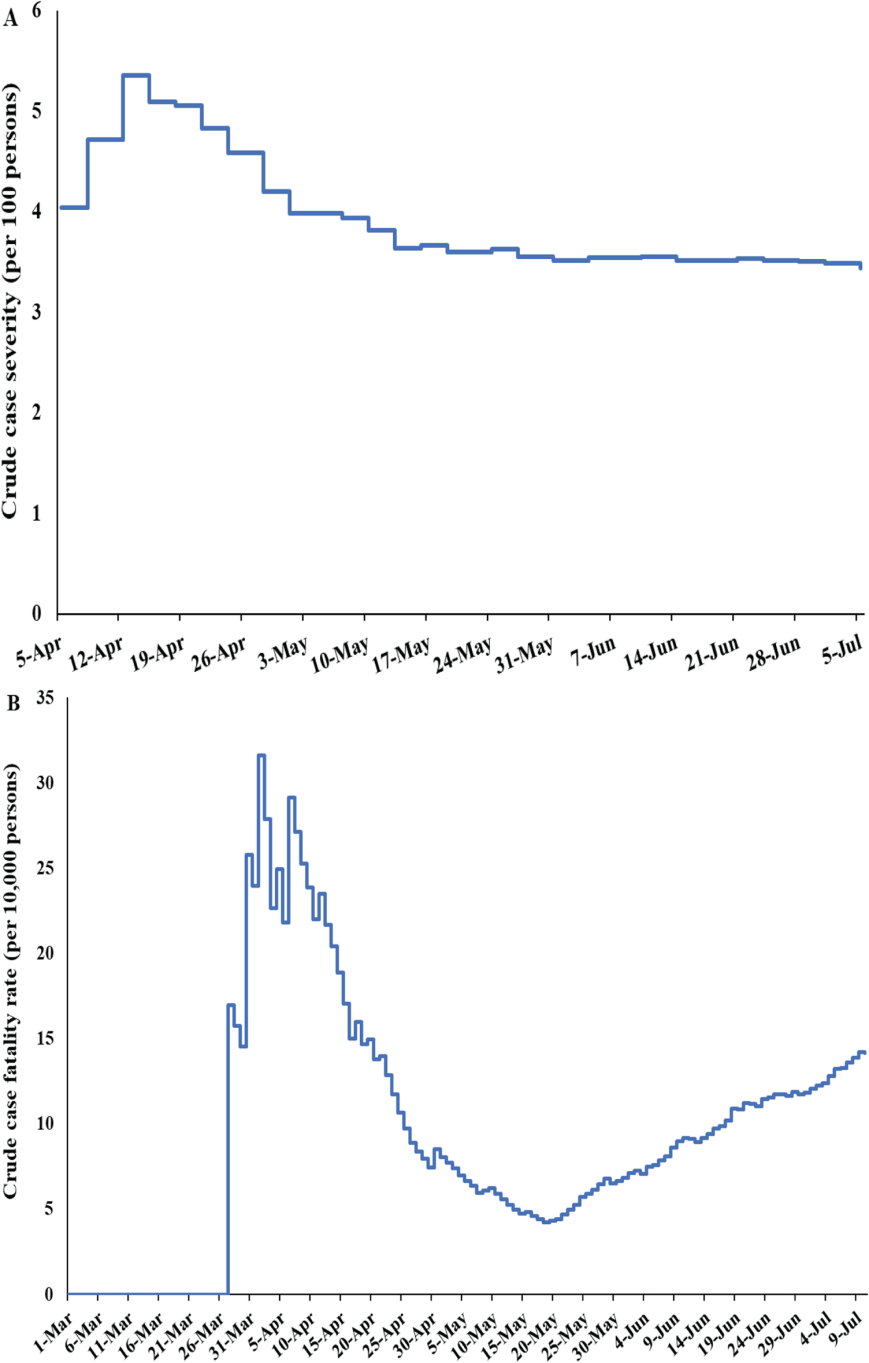
Temporal trend in (**A**) crude case severity rate and (**B**) crude case fatality rate in Qatar.

Figure 3A shows the crude case severity rate versus time defined as the cumulative number of severe and critical infections over the cumulative number of laboratory-confirmed infections. The crude case severity rate was mostly stable, assessed at 3.4% on July 5.

Figures 3B shows the crude case fatality rate versus time defined as the cumulative number of COVID-19 deaths over the cumulative number of laboratory-confirmed infections. The crude case fatality rate varied throughout the epidemic, in part because of the age structure of the population affected at each time point, but mostly because of statistical volatility with the relatively small number of COVID-19 deaths (146 as of July 10). The crude case fatality rate has been increasing steadily but slowly in recent weeks, consistent with the epidemic dynamics moving from the labor to the urban population where virtually all the elderly population resides. As of July 10, the crude case fatality rate was 0.14%.

## Discussion

The SARS-CoV-2 epidemic in Qatar was investigated using different lines of epidemiological evidence and study methodologies, utilizing a centralized and standardized national data-capture system and a national response that emphasized broad testing and provision of early, rapid, standardized, and universal healthcare. Strikingly, these independent lines of evidence converged on similar and consistent findings: Qatar has experienced a pervasive but heterogeneous SARS-CoV-2 epidemic, that is already declining rapidly, apparently due to rising level of immunity in the population. The national epidemic implicitly included two linked and overlapping but different sub-epidemics. The first affected the labor population, which constitutes the majority of Qatar’s population, and grew rapidly before peaking and then starting to decline. The second and slowly growing sub-epidemic affected the urban population, and appears to be plateauing if not declining slowly, with potential for growth if the easing of the social and physical distancing restrictions proceeds too quickly.

Based on our understanding of the global epidemiology of the SARS-CoV-2 infection^24^ and as informed by country-specific studies^13,25–27^, the epidemic in Qatar appears to be one of the most advanced worldwide as about half of the population has already been exposed to the infection^28–31^. This contrasts with the experience of other countries and global regions where those exposed have been estimated to account, by WHO region, for 0.7% of the population in the Western Pacific Region, 3.3% in the African Region, 8.1% in the South-East Asia Region, and 25.5% in the European Region^24^. The closest to the experience of Qatar was that of the Americas region where exposure has been estimated at 41.9%^24^.

Epidemic intensity in Qatar reflected the unique demographic and residential dwelling structure in this country, where the majority of the population live in shared housing. The most affected subpopulation was that of the majority population of single CMW living in shared housing accommodations, where workers at a given workplace not only work together during the day, but also typically live together in large dormitories where they share rooms, bathrooms, and cafeteria-style meals. In these settings, the pattern of SARS-CoV-2 transmission showed resemblance to that of influenza outbreaks in schools^32,33^, and more so to boarding schools^33^, where the options for effective social and physical distancing are reduced. The differences in exposure by nationality reflected the contribution of each nationality to the labor versus urban population. Yet, these differences may also reflect the structure of social networks in Qatar, as social contacts could be higher among groups who share the same culture, language, and/or national background.

Remarkably, while widespread, the infection has been characterized by relatively low case severity and fatality rates (Fig. 3). The young age profile of the population, with only 8.8% being > 50 years of age, appears to explain part of the low severity—88.4% of confirmed infections were among those < 50 years of age. The fact that the epidemic was most intense in the young and healthy CMW population, as opposed to the urban population where all elderly reside, contributed also to the low severity. Indeed, analyses indicated a strong role for age in disease severity and mortality (Fig. 2), with even higher effect sizes than elsewhere^34–37^, possibly because of greater accounting of asymptomatic infection in Qatar.

The resourced healthcare system, which was well below the health system threshold even at the epidemic peak, may have also contributed to the low mortality. Emphasis on broad testing coupled with proactive early treatment, such as the treatment of > 4,000 cases for pneumonia, may have limited the number of people who went on to require hospitalization or to develop severe or critical disease.

A notable feature of the epidemic, that is also possibly linked to the population’s young demographic structure, is the large proportion of infections that were asymptomatic or with minimal/mild symptoms for infection to be suspected. Out of every five identified infections, three were diagnosed at a health facility, somewhat a proxy for symptomatic infection, while the other two were diagnosed through contact tracing or surveillance testing in workplaces or residential areas. A large fraction of PCR positive individuals in the community survey (~ 60%) were also asymptomatic with relatively high PCR Ct value, suggesting that these individuals were probably in an advanced infection stage and thus not likely to develop symptoms following the survey date^23^. Hospital and isolation facility records show that none of these asymptomatically-infected persons were admitted subsequently with severe or critical disease as of July 10, but there is record indicating that two of them (out of 72) did develop subsequently a symptomatic mild infection.

Meanwhile, 20.5% of those PCR negative in the community survey reported at least one symptom, suggesting that among those who were positive *and* symptomatic, some symptoms may not have been related to SARS-CoV-2 infection (Table 3). Notably, presence of one or two symptoms was not predictive of infection, but presence of three or more symptoms was strongly predictive (Table 3). Although several symptoms were associated (and strongly) with infection (Supplementary Table S2), very few infected persons reported them (< 10%), apart from fever which was reported by 29% of infected persons.

Conduct of the series of epidemiological studies reported here was commissioned by the Ministry of Public Health in Qatar and the results and findings played an influential role in informing the national public-health response and in formulating evidence-based policy decisions that minimized the epidemic’s toll on health, society, and the economy. While this article illustrates a successful application to inform the national response, this epidemiological approach in generating strategic data about the epidemic can be adapted and applied in other countries to guide SARS-CoV-2 epidemic control as well as preparedness for the current or future infection waves.

## Limitations

This study has limitations. The community survey questionnaire was administered only in two main languages, Arabic and English, in this multilingual community, which may have introduced bias to reported answers. Symptoms in this survey were based on self-report, thus also introducing potential for recall bias. Although the sampling was intended to be probability-based, most participants were recruited through convenience sampling due to poor response rate suggesting potential for selection bias. However, there was no evidence that PCR positivity differed by method of recruitment (Table 2). Moreover, observed PCR prevalence was similar in both the community survey and the ad-hoc testing campaigns conducted in diverse workplaces and residential areas (Table 2 and Supplementary Table S4). Analyses of predictors of severity and mortality were limited in scope, not accounting for relevant covariates, such as comorbidities. COVID-19 mortality was based on confirmed hospital deaths and out of hospital deaths per WHO classification^3^, but such mortality registry may not capture excess deaths caused indirectly by this infection, or deaths misclassified for other causes. Study outcomes may be affected by the sensitivity or specificity of the assays used. However, laboratory methods were based on quality commercial platforms, and each diagnostic method was validated in the laboratory before its use. All results, regardless of the laboratory method used, were also consistent with each other, and specificity of the antibody assay, even if not perfect, may not affect the results given the high antibody prevalence. Of note that the specificity of the antibody assay was reported at 99.8%^15^ by the manufacturer and at 100% by a validation study by Public Health England^38^. Remarkably, among those who were diagnosed PCR positive or negative > 3 weeks before being tested for detectable antibodies (the three weeks to allow for antibodies to be detectable^21^), the percent agreement between the PCR outcome and the antibody outcome was 94.4%, affirming the consistency of both the PCR and antibody methods in diagnosing infection.

## Conclusions

In conclusion, we provided a detailed descriptive epidemiology of the SARS-CoV-2 epidemic in Qatar by answering specific research questions through conduct of a series of national epidemiologic studies. Our multi-faceted characterization of the epidemic in this country provided broader inferences about the epidemiology of this infection. First, SARS-CoV-2 is a highly infectious virus with a large basic reproduction number *R*_*0*_, that is considerably larger than that for other typical cold respiratory viruses^39–41^. Despite the enforced restrictive social and physical distancing measures, *R*_*0*_ must have been well above 1 to lead to such large epidemic. Second, while age has been recognized as an important factor since the beginning of the pandemic^34–37,42,43^. it appeared to play even a more critical role in the epidemiology in Qatar than estimated thus far. Not only were serious disease and mortality very strongly linked to being > 50 years of age, but most infections in younger persons exhibited no or minimal/mild symptoms. Age may have even played a role in the risk of exposure or susceptibility to the infection (Table 5), as suggested earlier^44,45^. These findings may suggest that the epidemic expansion in nations with young populations may lead to milder disease burden than previously thought.

## Supplementary Information

Supplementary Information
