# Supplementary material for: Characterizing the Qatar advanced-phase SARS-CoV-2 epidemic
Source: Sci Rep. 2021 Mar 18;11:6233. doi: 10.1038/s41598-021-85428-7 (PMC7973743; doi:10.1038/s41598-021-85428-7)
Supplement: Supplementary file 1 — Supplementary Information [file 41598_2021_85428_MOESM1_ESM.pdf]

Supplementary material

Characterizing the Qatar advanced-phase SARS-CoV-2 epidemic

Laith J. Abu Raddad,[1,2,3*] Hiam Chemaitelly,[1,2] Houssein H. Ayoub,[4] Zaina Al Kanaani,[5] Abdullatif Al Khal,[5] Einas Al Kuwari,[5] Adeel A. Butt,[5] Peter Coyle,[5] Andrew Jeremijenko,[5] Anvar Hassan Kaleeckal,[5] Ali Nizar Latif,[5] Robert C. Owen,[5] Hanan F. Abdul Rahim,[6] Samya A. Al Abdulla,[7] Mohamed G. Al Kuwari,[7] Mujeeb C. Kandy,[7] Hatoun Saeb,[7] Shazia Nadeem N. Ahmed,[8] Hamad Eid Al Romaihi,[8] Devendra Bansal,[8] Louise Dalton,[8] Sheikh Mohammad Al Thani,[8] and Roberto Bertollini[8]

[1]*Infectious Disease Epidemiology Group, Weill Cornell Medicine-Qatar, Cornell University, Doha, Qatar*

[2]*World Health Organization Collaborating Centre for Disease Epidemiology Analytics on HIV/AIDS, Sexually Transmitted Infections, and Viral Hepatitis, Weill Cornell Medicine–Qatar, Cornell University, Qatar Foundation – Education City, Doha, Qatar*

[3]*Department of Population Health Sciences, Weill Cornell Medicine, Cornell University, New York, New York, USA*

[4]*Department of Mathematics, Statistics, and Physics, Qatar University, Doha, Qatar*

[5]*Hamad Medical Corporation, Doha, Qatar*[6]*College of Health Sciences, QU Health, Qatar University, Doha, Qatar*

[7]*Primary Health Care Corporation, Doha, Qatar*

[8]*Ministry of Public Health, Doha, Qatar*

Address reprints requests or correspondence to Professor Laith J. Abu-Raddad, Infectious Disease Epidemiology Group, World Health Organization Collaborating Centre for Disease Epidemiology Analytics on HIV/AIDS, Sexually Transmitted Infections, and Viral Hepatitis, Weill Cornell Medicine - Qatar, Qatar Foundation - Education City, P.O. Box 24144, Doha, Qatar. Telephone: +(974) 4492-8321. Fax: +(974) 4492-8333. E-mail: lja2002@qatar-med.cornell.edu.

Supplementary Text S1. SARS-CoV-2 population-based survey questionnaire.

Qatar National Identification Number

**1. Data Collector Information**	
Form completion date (DD/MM/YYYY)	___/_ /____
Primary Health Care Corporation Center	
**2. Identifier information**	
First name	
Surname	
Sex	□ Male □ Female □ Not known
Date of Birth (DD/MM/YYYY)	___/_ /____
Telephone (mobile) number	
Age (years)	
Nationality	
Education	
Occupation	
Have you had contact with anyone with suspected or confirmed COVID-19 virus infection?	□ Yes □ No □ Unknown<br><br>If Yes, dates of last contact (DD/MM/YYYY):<br>___/_ /____
**3. Symptom history**	
In the past TWO WEEKS, have you had any of the following:	
Fever ≥38°C	□ Yes □ No
Chills	□ Yes □ No
Fatigue	□ Yes □ No
Muscle ache (myalgia)	□ Yes □ No
Sore throat	□ Yes □ No
Cough	□ Yes □ No
Runny nose (rhinorrea)	□ Yes □ No
Shortness of breath (dyspnea)	□ Yes □ No
Wheezing	□ Yes □ No
Chest pain	□ Yes □ No
Other respiratory symptoms	□ Yes □ No
Headache	□ Yes □ No
Nausea/vomiting	□ Yes □ No
Abdominal pain	□ Yes □ No
Diarrhea	□ Yes □ No
Loss of sense of smell	□ Yes □ No
Loss of sense of taste	□ Yes □ No
Did any of these symptoms require you to seek medical attention?	□ Yes □ No □ Unknown
Did any of these symptoms require you to be hospitalized?	□ Yes □ No □ Unknown

Supplementary Table S1. Socio-demographic characteristics of participants in the community survey conducted on May 6-7, 2020 with respect to study recruitment/invitation status.

**Socio-demographic characteristics**	**Invited (probability-based sampling; sample=268)**	**Not invited (convenience sampling; sample=687)**	
	N (%*)	N (%*)	p-value
Sex			
Males	216 (80.6)	559 (81.4)	0.784
Females	52 (19.4)	128 (18.6)	
Age (years)			
<30	36 (13.4)	184 (26.8)	<0.001
30-39	88 (32.8)	302 (44.0)	
40-49	64 (23.9)	125 (18.2)	
50+	80 (29.9)	76 (11.1)	
Nationality			
Other[illegible]	68 (25.4)	139 (20.2)	<0.001
Indian	78 (29.1)	273 (39.7)	
Filipino	7 (2.6)	58 (8.4)	
Sri Lankan	3 (1.1)	18 (2.6)	
Egyptian	30 (11.2)	52 (7.6)	
Nepalese	6 (2.2)	12 (1.8)	
Bangladeshi	2 (0.8)	7 (1.0)	
Pakistani	9 (3.4)	9 (1.3)	
Sudanese	6 (2.2)	10 (1.5)	
Qatari	59 (22.0)	109 (15.9)	
Educational attainment			
Bachelor's degree	129 (48.1)	291 (42.4)	0.001
High school	90 (33.6)	282 (41.1)	
Post-graduate studies	41 (15.3)	63 (9.2)	
Unknown	8 (3.0)	51 (7.4)	
Occupation			
Healthcare	15 (5.7)	30 (4.4)	0.217
Aviation/Police/Government	18 (6.8)	34 (5.0)	
Hospitality/Retail/Education	33 (12.5)	66 (9.7)	
Professional/Office	73 (27.6)	191 (27.9)	
Transportation/driver	15 (5.7)	62 (9.1)	
Construction/Blue collar jobs	53 (20.0)	114 (16.7)	
Not employed	29 (10.9)	88 (12.9)	
Unknown/Other	29 (10.9)	99 (14.5)	

*Column percentages.

Supplementary Table S2. Reported symptoms by individuals tested for SARS-CoV-2 by PCR during the community survey conducted on May 6-7, 2020 and associations with current infection.

**Characteristics**	**Sample size[*]**	**Sample with confirmed test results**	**SARS-CoV-2 positive[†]**		**Univariable regression analysis**	
	N (%)	N (%[‡])	N (%[§])	p-value	OR (95% CI)[¶]	p-value
Fever						
No	1,063 (93.6)	1,045 (92.4)	100 (11.7)	<0.001	1.00	
Yes	73 (6.4)	69 (7.6)	33 (56.8)		9.93 (4.06-24.29)	<0.001
Chills						
No	1,131 (99.6)	1,109 (99.4)	132 (15.1)	0.772	1.00	
Yes	5 (0.4)	5 (0.6)	1 (11.2)		0.71 (0.06-7.62)	0.774
Fatigue						
No	1,115 (98.1)	1,095 (97.7)	126 (14.0)	<0.001	1.00	
Yes	21 (1.9)	19 (2.3)	7 (62.5)		10.22 (2.17-48.11)	0.003
Muscle ache						
No	1,109 (97.6)	1,090 (98.5)	125 (14.8)	0.016	1.00	
Yes	27 (2.4)	24 (1.5)	8 (35.7)		3.20 (1.18-8.64)	0.022
Sore throat						
No	1,035 (91.1)	1,017 (91.9)	110 (14.5)	0.195	1.00	
Yes	101 (8.9)	97 (8.1)	23 (22.1)		1.68 (0.76-3.69)	0.199
Cough						
No	1,019 (89.7)	1,003 (91.0)	103 (14.5)	0.183	1.00	
Yes	117 (10.3)	111 (9.0)	30 (21.5)		1.62 (0.79-3.31)	0.186
Runny nose						
No	1,091 (96.0)	1,070 (96.0)	129 (14.5)	0.249	1.00	
Yes	45 (4.0)	44 (4.0)	4 (30.5)		2.60 (0.48-13.91)	0.265
Shortness of breath						
No	1,111 (97.8)	1,090 (98.5)	129 (15.1)	0.981	1.00	
Yes	25 (2.2)	24 (1.5)	4 (15.3)		1.01 (0.32-3.20)	0.981
Wheezing						
No	1,129 (99.4)	1,107 (99.2)	132 (15.2)	0.596	1.00	
Yes	7 (0.6)	7 (0.8)	1 (8.9)		0.55 (0.06-5.28)	0.602
Chest pain						
No	1,110 (97.7)	1,089 (98.4)	125 (14.9)	0.140	1.00	
Yes	26 (2.3)	25 (1.6)	8 (25.9)		1.99 (0.78-5.07)	0.147
Other respiratory symptoms						
No	1,131 (99.6)	1,109 (99.7)	130 (14.9)	0.002	1.00	
Yes	5 (0.4)	5 (0.3)	3 (64.8)		10.49 (1.65-66.68)	0.013
Headache						
No	1,054 (92.8)	1,037 (93.9)	117 (14.1)	0.083	1.00	
Yes	82 (7.2)	77 (6.1)	16 (30.6)		2.69 (0.84-8.55)	0.094
Nausea/Vomiting						
No	1,130 (99.5)	1,108 (98.6)	130 (14.2)	<0.001	1.00	
Yes	6 (0.5)	6 (1.4)	3 (81.8)		27.17 (3.08-239.45)	0.003
Abdominal pain						
No	1,128 (99.3)	1,106 (99.5)	130 (15.0)	0.047	1.00	
Yes	8 (0.7)	8 (0.5)	3 (42.5)		4.19 (0.90-19.50)	0.068
Diarrhea						
No	1,126 (99.1)	1,104 (99.3)	130 (15.0)	0.092	1.00	
Yes	10 (0.9)	10 (0.7)	3 (36.1)		3.21 (0.77-13.48)	0.111
Loss of sense of smell						

No	1,120 (98.6)	1,103 (99.2)	128 (14.7)	<0.001	1.00	
Yes	16 (1.4)	11 (0.8)	5 (67.2)		11.91 (3.22-44.0)	<0.001
Loss of sense of taste						
No	1,126 (99.1)	1,107 (98.6)	128 (14.0)	<0.001	1.00	
Yes	10 (0.9)	7 (1.4)	5 (9.4)		104.01 (13.38-809.54)	<0.001

Abbreviations: CI, confidence interval. OR, odds ratio.
[*]Include 1,136 samples with information on symptoms
[†]Only 1,114 samples with information on symptoms and confirmed results were analyzed.
[‡]Weighted percentages by age and nationality.
[§]Row percentages weighted by age and nationality.
[¶]Estimates are weighted by age and nationality.

Supplementary Table S3. Association of PCR Ct values with presence of symptoms for the SARS-CoV-2 confirmed cases identified during the community survey conducted on May 6-7, 2020.

Target protein/gene	Ct value				Ct value <25		Univariable regression analysis	
	N	Mean	Median	Range	N (%*)	p-value	OR (95% CI)*	p-value
N protein†								
Asymptomatic	49	27.5	27.5	16.8-35.7	21 (36.7)	0.041	1.00	
Symptomatic	37	21.0	21.0	15.2-33.0	26 (73.3)		4.72 (1.02-21.95)	0.048
S protein†								
Asymptomatic	48	26.8	28.5	16.7-37.0	20 (35.8)	0.037	1.00	
Symptomatic	37	22.5	22.4	15.4-32.5	26 (73.3)		4.92 (1.05-23.08)	0.044
ORF1ab†								
Asymptomatic	48	26.1	28.0	16.4-36.0	21 (37.0)	0.036	1.00	
Symptomatic	37	21.9	20.6	15.2-33.3	27 (74.5)		4.97 (1.05-23.51)	0.043
E gene‡								
Asymptomatic	22	29.2	30.6	19.5-36.3	6 (20.5)	0.039	1.00	
Symptomatic	21	26.5	26.3	17.1-35.8	9 (64.7)		7.07 (1.02-49.19)	0.048
All samples regardless of target protein or gene§								
Asymptomatic	71	27.2	28.7	16.8-36.3	27 (32.2)	0.008	1.00	
Symptomatic	58	23.8	23.2	15.2-35.8	58 (69.9)		4.89 (1.47-16.34)	0.010

Abbreviations: CI, confidence interval. OR, odds ratio. PCR, polymerase chain reaction.
*Estimates are weighted by age and nationality.
†Target assayed using the TaqPath™ COVID-19 Combo Kit (Thermo Fisher Scientific, USA).
‡Target assayed using the AccuPower SARS-CoV-2 Real-Time RT-PCR Kit (Bioneer, Korea).
§Ct values for N protein were used, and complemented by Ct values for E-gene in case of missing values. All individuals with Ct values for S protein or ORF1ab protein also had values for N protein.

Supplementary Table S4. Meta-analysis results for the pooled mean SARS-CoV-2 PCR positivity in the random testing campaigns conducted in workplaces and in residential areas, up to June 4, 2020.

	**Population groups**	**Sample**		**SARS-CoV-2 PCR testing positivity (%)**				**Heterogeneity measures**		
	Total N[a]	Tested	Positive	Median[*]	Range[*]	Pooled mean	95% CI	Q[†] (p-value)	$I^{2‡}$ (%, 95% CI)	Prediction interval[§] (95%)
Swab type										
Workplace	347	22,834	4,359	10.3	0.0-100.0	15.1	13.0-17.3	6494.8 (p<0.001)	94.7 (94.3-95.0)	0.0-64.2
Residential	45	3,881	834	19.0	0.0-81.5	20.3	15.4-25.6	563.3 (p<0.001)	92.2 (90.4-93.6)	0.0-58.0)
Time										
March[¶]	4	552	115	17.2	11.1-36.4	17.6	9.3-27.7	9.7 (p=0.022)	68.9 (10.2-89.3)	0.0-62.8
April 4-10	32	1,856	128	3.1	0.0-70.6	4.9	1.9-8.9	284.5 (p<0.001)	89.1 (85.7-91.7)	0.0-35.6
April 11-17	28	2,476	342	5.1	0.0-59.5	10.2	4.7-17.2	628.0 (p<0.001)	95.7 (94.6-96.5)	0.0-58.7
April 18-24	56	5,422	1,130	18.0	0.0-71.1	19.7	14.2-25.9	1456.2 (p<0.001)	96.2 (95.6-96.7)	0.0-70.7
April 25-May 01	39	2,548	568	21.6	0.0-81.8	21.5	14.8-29.1	659.2 (p<0.001)	94.2 (92.9-95.3)	0.0-72.5
May 02-08	65	4,254	838	12.1	0.0-69.8	14.9	10.9-19.4	849.0 (p<0.001)	92.5 (91.1-93.6)	0.0-56.5
May 09-15	36	3,189	837	23.0	0.0-85.4	23.1	15.6-31.5	909.6 (p<0.001)	96.2 (95.4-96.8)	0.0-77.2
May 16-22	56	3,103	829	21.8	0.0-100.0	23.0	16.8-29.8	892.2 (p<0.001)	93.8 (92.7-94.8)	0.0-75.9
May 23-29	48	2,175	362	13.4	0.0-87.9	15.8	10.6-21.7	471.2 (p<0.001)	90.0 (87.6-92.0)	0.0-61.2
May 30-June 04	28	1,140	44	1.5	0.0-21.7	2.5	1.0-4.5	57.8 (p<0.001)	53.2 (28.1-69.6)	0.0-12.7
**Overall**	**392**	**26,715**	**5,193**	**11.5**	**0.0-100.0**	**15.6**	**13.7-17.7**	**7101.2 (p<0.001)**	**94.5 (94.1-94.8)**	**0.0-63.1**

[*]Testing campaign sample with sample size <10 were grouped together or with the testing campaign sample that has the lowest sample size ≥10
[†]Q: the Cochran's Q statistic is a measure assessing the existence of heterogeneity in effect size (here, SARS-CoV-2 PCR prevalence) across studies.
[‡]I^2: a measure assessing the magnitude of between-study variation that is due to differences in effect size (here, SARS-CoV-2 PCR prevalence) across studies rather than chance.
[§]Prediction interval: a measure estimating the 95% interval of the distribution of true effect sizes (here, SARS-CoV-2 PCR prevalence measures).
[¶]Exact dates were not available.

Supplementary Table S5. Basic associations with SARS-CoV-2 severe infection in Qatar based on analysis of the national SARS-CoV-2 PCR testing and hospitalization database. Severe infection was based on WHO classification.[1]

Characteristics	Sample size	SARS-CoV-2 severe infection		Univariable analysis		Multivariable analysis	
	N (%)	N (%)	p-value	OR (95% CI)	p-value*	AOR (95% CI)	p-value†
Sex							
Females	13,572 (14.2)	229 (1.7)	<0.001	1.00		1.00	
Males	82,175 (85.8)	2,207 (2.7)		1.61 (1.40-1.85)	<0.001	1.58 (1.35-1.84)	<0.001
Age (years)							
0-29	30,208 (31.5)	92 (0.3)	<0.001	1.00		1.00	
30-39	34,487 (36)	609 (1.8)		5.89 (4.73-7.34)	<0.001	5.57 (4.47-6.95)	<0.001
40-49	19,826 (20.7)	750 (3.8)		12.96 (10.43-16.10)	<0.001	12.76 (10.25-15.88)	<0.001
50-59	8,333 (8.7)	562 (6.9)		24.13 (19.33-30.13)	<0.001	25.84 (20.66-32.32)	<0.001
60-69	2,342 (2.4)	316 (14.1)		53.68 (42.37-68.02)	<0.001	59.15 (46.53-75.18)	<0.001
70-79	440 (0.5)	84 (20.8)		86.16 (62.83-118.14)	<0.001	98.24 (70.87-136.17)	<0.001
80+	111 (0.1)	23 (25.8)		114.02 (68.00-191.18)	<0.001	134.99 (78.85-231.11)	<0.001
Nationality							
All other nationalities‡	9,501 (9.9)	239 (2.5)	<0.001	1.00		1.00	
Indian	28,111 (29.4)	450 (1.6)		0.63 (0.54-0.74)	<0.001	0.47 (0.39-0.55)	<0.001
Bangladeshi	13,604 (14.2)	532 (3.9)		1.58 (1.35-1.85)	<0.001	1.42 (1.20-1.68)	<0.001
Nepalese	17,548 (18.3)	420 (2.4)		0.95 (0.81-1.11)	0.517	1.06 (0.89-1.26)	0.531
Pakistani	5,967 (6.2)	218 (3.7)		1.48 (1.23-1.78)	<0.001	1.13 (0.93-1.38)	0.209
Sudanese	1,963 (2.1)	38 (1.9)		0.76 (0.54-1.08)	0.129	0.57 (0.40-0.81)	0.002
Sri Lankan	3,433 (3.6)	79 (2.3)		0.91 (0.70-1.18)	0.473	0.76 (0.58-0.99)	0.042
Egyptian	4,468 (4.7)	125 (2.8)		1.11 (0.89-1.39)	0.333	0.86 (0.69-1.08)	0.207
Filipino	5,116 (5.3)	207 (4.1)		1.64 (1.36-1.99)	<0.001	1.35 (1.11-1.65)	0.003
Qatari	6,036 (6.3)	128 (2.1)		0.84 (0.68-1.04)	0.112	0.82 (0.65-1.04)	0.101
Time							
05 Feb-31 March	1,427 (1.5)	44 (3.1)	<0.001	1.00		1.00	
01-15 April	3,270 (3.4)	134 (4.2)		1.33 (0.94-1.89)	0.104	1.75 (1.22-2.50)	0.002
16-30 April	9,732 (10.2)	272 (2.8)		0.89 (0.65-1.23)	0.494	1.26 (0.91-1.77)	0.169
01-15 May	17,221 (18.0)	483 (2.8)		0.89 (0.65-1.22)	0.486	1.28 (0.93-1.77)	0.137
16-31 May	27,692 (28.9)	812 (3.0)		0.94 (0.69-1.27)	0.671	1.27 (0.92-1.74)	0.143
01-15 June	23,838 (24.9)	640 (2.7)		0.85 (0.62-1.16)	0.311	1.17 (0.85-1.61)	0.348
16-24 June	12,567 (13.1)	51 (0.4)		0.13 (0.08-0.19)	<0.001	0.17 (0.11-0.26)	<0.001

Abbreviations: AOR, adjusted odds ratio. CI, confidence interval. OR, odds ratio. PCR, polymerase chain reaction.
*Covariates with p-value ≤0.1 in the univariable analysis were included in the multivariable analysis.
†Covariates with p-value ≤0.05 in the multivariable analysis were considered predictors of SARS-CoV-2 severe infection.
‡These include all other nationalities residing in Qatar.

Supplementary Table S6. Basic associations with SARS-CoV-2 critical infection in Qatar based on analysis of the national SARS-CoV-2 PCR testing and hospitalization database. Critical infection was based on WHO classification.[1]

Characteristics	Sample size	SARS-CoV-2 critical infection		Univariable analysis		Multivariable analysis	
	N (%)	N (%)	p-value	OR (95% CI)	p-value*	AOR (95% CI)	p-value†
Sex							
Females	13,572 (14.2)	52 (0.4)	0.002	1.00		1.00	
Males	82,175 (85.8)	487 (0.6)		1.57 (1.18-2.09)	0.002	1.58 (1.16-2.16)	0.004
Age (years)							
0-29	30,208 (31.5)	14 (0.05)	<0.001	1.00		1.00	
30-39	34,487 (36.0)	76 (0.2)		4.83 (2.73-8.55)	<0.001	4.30 (2.43-7.61)	<0.001
40-49	19,826 (20.7)	138 (0.7)		15.67 (9.04-27.16)	<0.001	14.26 (8.21-24.77)	<0.001
50-59	8,333 (8.7)	152 (2.0)		42.90 (24.80-74.20)	<0.001	42.17 (24.30-73.19)	<0.001
60-69	2,342 (2.4)	100 (4.9)		111.64 (63.69-195.67)	<0.001	117.78 (66.86-207.49)	<0.001
70-79	440 (0.5)	37 (10.4)		249.39 (133.51-465.83)	<0.001	298.20 (156.62-567.77)	<0.001
80+	111 (0.1)	22 (25.0)		716.71 (351.57-1461.10)	<0.001	1163.70 (544.87-2485.34)	<0.001
Nationality							
All other nationalities‡	9,501 (9.9)	55 (0.6)	<0.001	1.00		1.00	
Indian	28,111 (29.4)	119 (0.4)		0.72 (0.52-1.00)	0.048	0.69 (0.49-0.98)	0.040
Bangladeshi	13,604 (14.2)	107 (0.8)		1.38 (1.00-1.91)	0.052	1.69 (1.18-2.42)	0.004
Nepalese	17,548 (18.3)	65 (0.4)		0.64 (0.44-0.91)	0.014	1.12 (0.75-1.66)	0.578
Pakistani	5,967 (6.2)	62 (1.1)		1.83 (1.27-2.63)	0.001	1.36 (0.93-2.01)	0.116
Sudanese	1,963 (2.1)	11 (0.6)		0.96 (0.50-1.84)	0.907	0.88 (0.45-1.72)	0.708
Sri Lankan	3,433 (3.6)	10 (0.3)		0.50 (0.25-0.98)	0.045	0.60 (0.30-1.19)	0.145
Egyptian	4,468 (4.7)	22 (0.5)		0.85 (0.52-1.40)	0.527	0.83 (0.50-1.39)	0.484
Filipino	5,116 (5.3)	59 (1.2)		2.04 (1.41-2.95)	<0.001	2.36 (1.59-3.51)	<0.001
Qatari	6,036 (6.3)	29 (0.5)		0.83 (0.53-1.30)	0.405	0.63 (0.39-1.02)	0.059
Time							
05 Feb-31 March	1,427 (1.5)	29 (2.1)	<0.001	1.00		1.00	
01-15 April	3,270 (3.4)	43 (1.4)		0.65 (0.40-1.04)	0.075	0.95 (0.58-1.56)	0.839
16-30 April	9,732 (10.2)	90 (1.0)		0.45 (0.29-0.68)	<0.001	0.68 (0.44-1.06)	0.090
01-15 May	17,221 (18.0)	124 (0.7)		0.35 (0.23-0.52)	<0.001	0.53 (0.34-0.82)	0.004
16-31 May	27,692 (28.9)	169 (0.6)		0.30 (0.20-0.44)	<0.001	0.42 (0.28-0.64)	<0.001
01-15 June	23,838 (24.9)	78 (0.3)		0.16 (0.10-0.24)	<0.001	0.21 (0.14-0.34)	<0.001
16-24 June	12,567 (13.1)	6 (0.05)		0.02 (0.01-0.05)	<0.001	0.03 (0.01-0.07)	<0.001

Abbreviations: AOR, adjusted odds ratio. CI, confidence interval. OR, odds ratio.
Proportions were reported using one decimal place, except for those <0.1% which were reported using two decimal places.
*Covariates with p-value ≤0.1 in the univariable analysis were included in the multivariable analysis.
†Covariates with p-value ≤0.05 in the multivariable analysis were considered predictors of SARS-CoV-2 critical infection.
‡These include all other nationalities residing in Qatar.

Supplementary Table S7. Basic associations with COVID-19 death in Qatar based on analysis of the national COVID-19 mortality database. COVID-19 death was based on WHO classification.[2]

Characteristics	Total infections	Deaths		Univariable regression analysis		Multivariable regression analysis	
	N (%)	N (%)	p-value	OR (95% CI)	p-value*	AOR (95% CI)	p-value†
Sex							
Males	82,152 (85.8)	80 (0.1)	0.919	1.00		--	--
Females	13,548 (14.2)	13 (0.1)		0.99 (0.55-1.77)	0.961	--	--
Age (years)							
<50	84,471 (88.3)	16 (0.02)	<0.001	1.00		1.00	
50-59	8,335 (8.7)	30 (0.4)		19.07 (10.39-34.99)	<0.001	20.21 (10.88-37.55)	<0.001
60-69	2,343 (2.4)	23 (1.1)		52.33 (27.61-99.18)	<0.001	55.14 (28.21-107.80)	<0.001
70+	551 (0.6)	24 (5.0)		240.38 (126.96-455.13)	<0.001	309.37 (148.91-642.74)	<0.001
Nationality							
All other nationalities‡	9,489 (9.9)	10 (0.1)	<0.001	1.00		1.00	
Indian	28,105 (29.4)	4 (0.1)		0.30 (0.16-0.58)	<0.001	0.49 (0.24-0.99)	0.046
Bangladeshi	13,607 (14.2)	17 (0.1)		0.62 (0.32-1.20)	0.157	1.28 (0.63-2.62)	0.500
Nepalese	17,546 (18.3)	7 (0.04)		0.20 (0.08-0.47)	<0.001	0.87 (0.34-2.23)	0.770
Pakistani	5,958 (6.2)	15 (0.3)		1.26 (0.64-2.48)	0.507	0.86 (0.43-1.72)	0.661
Sudanese	1,964 (2.1)	5 (0.9)		0.76 (0.23-2.58)	0.663	0.80 (0.23-2.78)	0.721
Sri Lankan	3,433 (3.6)	17 (0.1)		1.00 (1.00-1.00)	--	1.00 (1.00-1.00)	--
Egyptian	4,458 (4.7)	10 (0.2)		0.11 (0.01-0.84)	0.033	0.14 (0.02-1.09)	0.060
Filipino	5,115 (5.3)	4 (0.9)		0.98 (0.45-2.10)	0.951	1.79 (0.79-4.07)	0.166
Qatari	6,025 (6.3)	4 (0.7)		0.33 (0.11-0.97)	0.045	0.2 (0.07-0.61)	0.004
Time							
05 Feb-31 March	1,425 (1.5)	8 (0.6)	<0.001	1.00		1.00	
01-15 April	3,265 (3.4)	4 (0.1)		0.22 (0.07-0.72)	0.013	0.31 (0.09-1.05)	0.061
16-30 April	9,733 (10.2)	10 (0.1)		0.18 (0.07-0.46)	<0.001	0.29 (0.11-0.77)	0.013
01-15 May	17,213 (18.0)	23 (0.1)		0.24 (0.11-0.53)	<0.001	0.38 (0.16-0.88)	0.024
16-31 May	27,689 (28.9)	41 (0.1)		0.26 (0.12-0.56)	0.001	0.38 (0.17-0.85)	0.018
01-15 June	23,817 (24.9)	7 (0.03)		0.05 (0.02-0.14)	<0.001	0.07 (0.03-0.20)	<0.001
16-24 June	12,558 (13.1)	0 (0.0)		1.00 (1.00-1.00)	--	1.00 (1.00-1.00)	--

Abbreviations: AOR, adjusted odds ratio. CI, confidence interval. OR, odds ratio.
Proportions were reported using one decimal place, except for those <0.1% which were reported using two decimal places.
*Covariates with p-value ≤0.1 in the univariable analysis were included in the multivariable analysis.
†Covariates with p-value ≤0.05 in the multivariable analysis were considered predictors of COVID-19 death.
‡These include all other nationalities residing in Qatar.

References

1 World Health Organization. Clinical management of COVID-19. Available from: https://www.who.int/publications-detail/clinical-management-of-covid-19. Accessed on: May 31st 2020. (2020).

2 World Health Organization. International guidelines for certification and classification (coding) of COVID-19 as cause of death. Available from: https://www.who.int/classifications/icd/Guidelines_Cause_of_Death_COVID-19-20200420-EN.pdf?ua=1. Document Number: WHO/HQ/DDI/DNA/CAT. Accessed on June 1, 2020. . (2020).
